# *Leishmania donovani* infection suppresses Allograft Inflammatory Factor-1 in monocytes and macrophages to inhibit inflammatory responses

**DOI:** 10.1038/s41598-020-79068-6

**Published:** 2021-01-13

**Authors:** Ricardo Louzada da Silva, Diana M. Elizondo, Nailah Z. D. Brandy, Naomi L. Haddock, Thomas A. Boddie, Laís Lima de Oliveira, Amélia Ribeiro de Jesus, Roque Pacheco de Almeida, Tatiana Rodrigues de Moura, Michael W. Lipscomb

**Affiliations:** 1 Department of Biology, Howard University, 415 College St. NW. EE Just Hall - Biology Building, Washington, DC 20059 USA; 2 Laboratory of Immunology and Molecular Biology, Federal University of Sergipe, Aracaju, Brazil; 3 Department of Pediatrics and Communicable Diseases, Univ of Michigan Medical School, Ann Arbor, MI USA; 4 Stanford Immunology, Stanford University, Stanford, CA USA

**Keywords:** Infection, Immune evasion, Parasitic infection, Monocytes and macrophages

## Abstract

Macrophages and monocytes are important for clearance of *Leishmania* infections. However, immune evasion tactics employed by the parasite results in suppressed inflammatory responses, marked by deficient macrophage functions and increased accumulation of monocytes. This results in an ineffective ability to clear parasite loads. Allograft Inflammatory Factor-1 (AIF1) is expressed in myeloid cells and serves to promote immune responses. However, AIF1 involvement in monocyte and macrophage functions during parasitic infections has not been explored. This study now shows that *Leishmania donovani* inhibits AIF1 expression in macrophages to block pro-inflammatory responses. Mice challenged with the parasite had markedly reduced AIF1 expression in splenic macrophages. Follow-up studies using in vitro approaches confirmed that *L. donovani* infection in macrophages suppresses AIF1 expression, which correlated with reduction in pro-inflammatory cytokine production and increased parasite load. Ectopic overexpression of AIF1 in macrophages provided protection from infection, marked by robust pro-inflammatory cytokine production and efficient pathogen clearance. Further investigations found that inhibiting AIF1 expression in bone marrow cells or monocytes impaired differentiation into functional macrophages. Collectively, results show that AIF1 is a critical regulatory component governing monocyte and macrophage immune functions and that *L. donovani* infection can suppress the gene as an immune evasion tactic.

## Introduction

*Leishmania* is a genus of intracellular parasites that infect, survive and proliferate in antigen presenting myeloid cells, particularly dendritic cells, monocytes and macrophages^1,2^. *Leishmania donovani* (*L. donovani*) and *Leishmania infantum* (*L.* *infantum*) species cause visceral leishmaniasis (VL), which is uniquely characterized by swelling of the spleen and liver, rapid weight loss and anemia. If left untreated, the disease can be fatal. Terminally differentiated myeloid cells are the principal mediators for effective parasite elimination during infections. However, infection of *L. donovani* or *L. infantum* have shown to suppress immune functions of monocytes and macrophages, which allows for both survival and propagation within the host cell^3^.

Bone marrow generated Ly6C^+^ monocytes are recruited to inflammatory sites during infection(s). A combination of toll like-receptor agonists and/or cytokine stimulation can then direct their differentiation into mature macrophages or Tip-DCs for effective pathogen clearance^4–6^. However, during *L. donovani* or *L. infantum* infections, macrophages and inflammatory monocytes can become preferential targets of the pathogen. Studies have also found that monocytes provide a greater permissiveness to parasite proliferation than dendritic cells or macrophages in infected tissues^7–9^. Infection by *Leishmania* can  result in excessive accumulation of Ly6C^+^ monocytes, with concomitant depressed numbers and anti-leshmanial activities of macrophages^7,10,11^.

Allograft Inflammatory Factor-1 (AIF1) is a calcium-binding protein that interacts with protein kinase C (PKC) to trigger downstream NFkB signaling cascades^12^. AIF1 is expressed in macrophages, microglial and dendritic cells to promote inflammation, antigen presentation and T cell polarization^13–19^. The gene is expressed in myeloid cell lineages across multiple species and tissue types^20^. In addition, AIF1 is important in migration, phagocytosis, proliferation, survival and plays an important role in pro-inflammatory activity of macrophages^15,21–23^. Although, studies have shown that M-CSF induces AIF1 expression in macrophages^24^, it is not known whether the gene plays a role in anti-*Leishmania* immunity.

Recent studies in hematopoietic stem cells revealed a critical role of AIF1 in differentiation of conventional and monocyte-derived dendritic cells^12^. However, no study has assessed the role of AIF1 in monocyte-to-macrophage conversion, nor whether a pathogen can exploit the gene to perturb differentiation of myeloid subsets during infections. This report now shows that *L. donovani* infection inhibits AIF1 expression to suppress macrophage-monocyte functions leading to restrained immune responses.

## Results

### AIF1 is expressed in macrophage-monocyte subsets of the spleen

**Figure 1:**
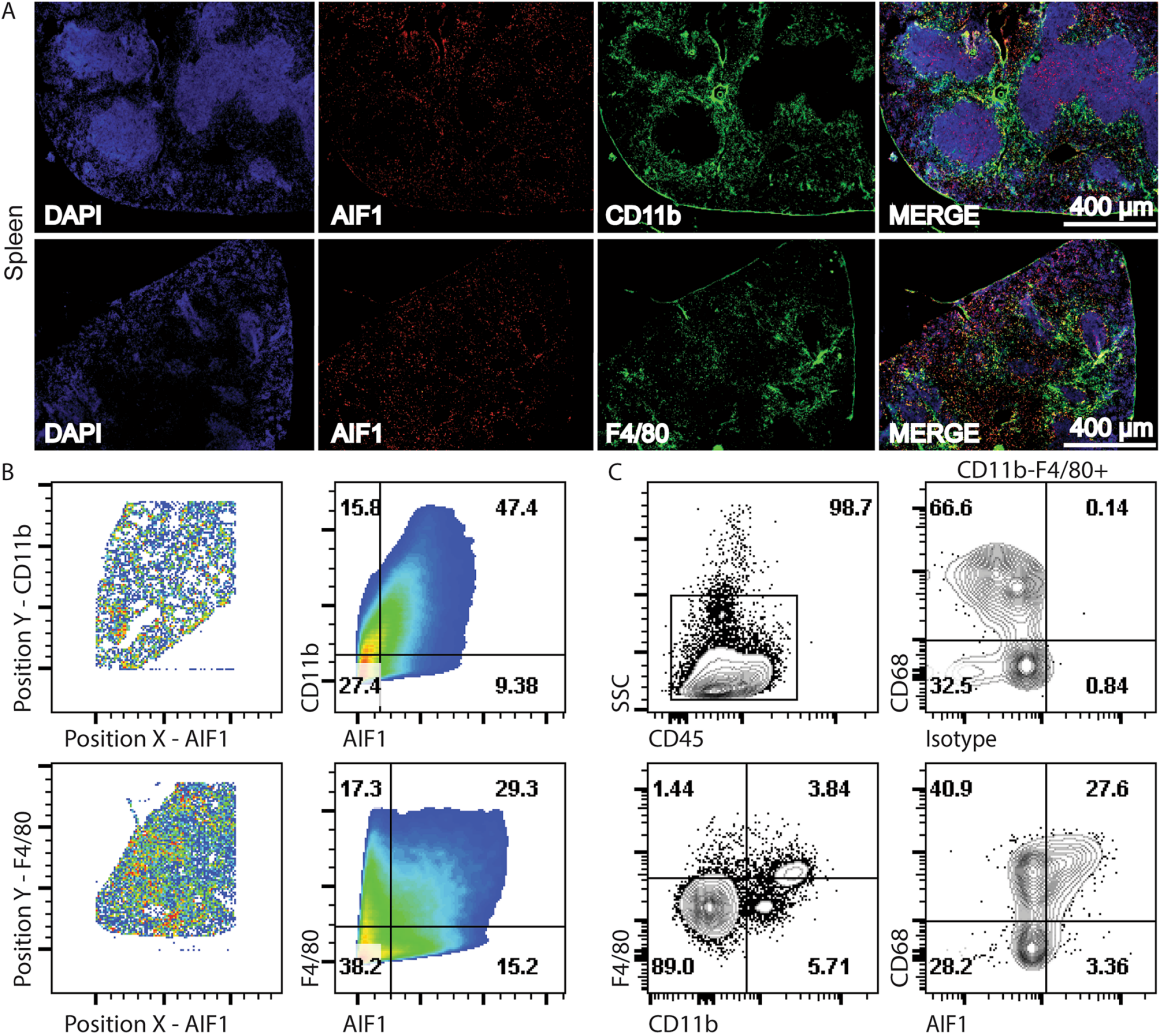
Tissue monocyte-macrophage lineages express AIF1. Spleen sections were prepared from C57BL6 mice (*n* = 6) to assess AIF1 expression by fluorescence microscopy and flow cytometry. (**A**) Tissues were cryosectioned at 16 μm and stained with fluorochrome conjugated antibodies to CD11b or F4/80 along with AIF1. DAPI dye was used for nuclear detection. Images were acquired using a standard wild-field fluorescence microscope and presented at 4x magnification. Microscopy data is one representative image of two independent experiments with a total of two mice. Scale bar represents 400 μm. (**B**) Images were further analyzed in FlowJo software for quantitative analyses. Co-localization of intensity of the Y-channel against the X-channel is plotted to determine co-expression of AIF1 with either CD11b or F4/80. (**C**) For flow cytometric analyses, spleen tissues were dissociated and stained with fluorochrome conjugated antibodies to CD11b, CD68, F4/80 and CD45 prior to acquisition. Cells were then analyzed for AIF1 and CD68 expression in CD11b^neg^F4/80^+^ subsets. Flow cytometric data is representative of four independent experiments.

Microscopy of mouse spleen sections revealed AIF1 co-localization in both CD11b^+^ and F4/80^+^ myeloid subsets, which comprise macrophage, dendritic cell and monocyte populations (Fig. 1A). Imaging datasets were next quantitatively assessed to determine AIF1 co-expression in the myeloid groups. Results revealed 47.4% co-localization of AIF1 with CD11b and 29.3% with F4/80 in splenocytes (Fig. 1B). Quantitation of microscopy data was corroborated by flow cytometric analyses of ex vivo dissociated tissues. Highest co-expression of AIF1 was found within the CD11b^lo/neg^F4/80^+^CD68^+^ splenic (red pulp) macrophages (Fig. 1C).

### *Leishmania* infection correlates with lowered AIF1 expression in splenic macrophages

**Figure 2:**
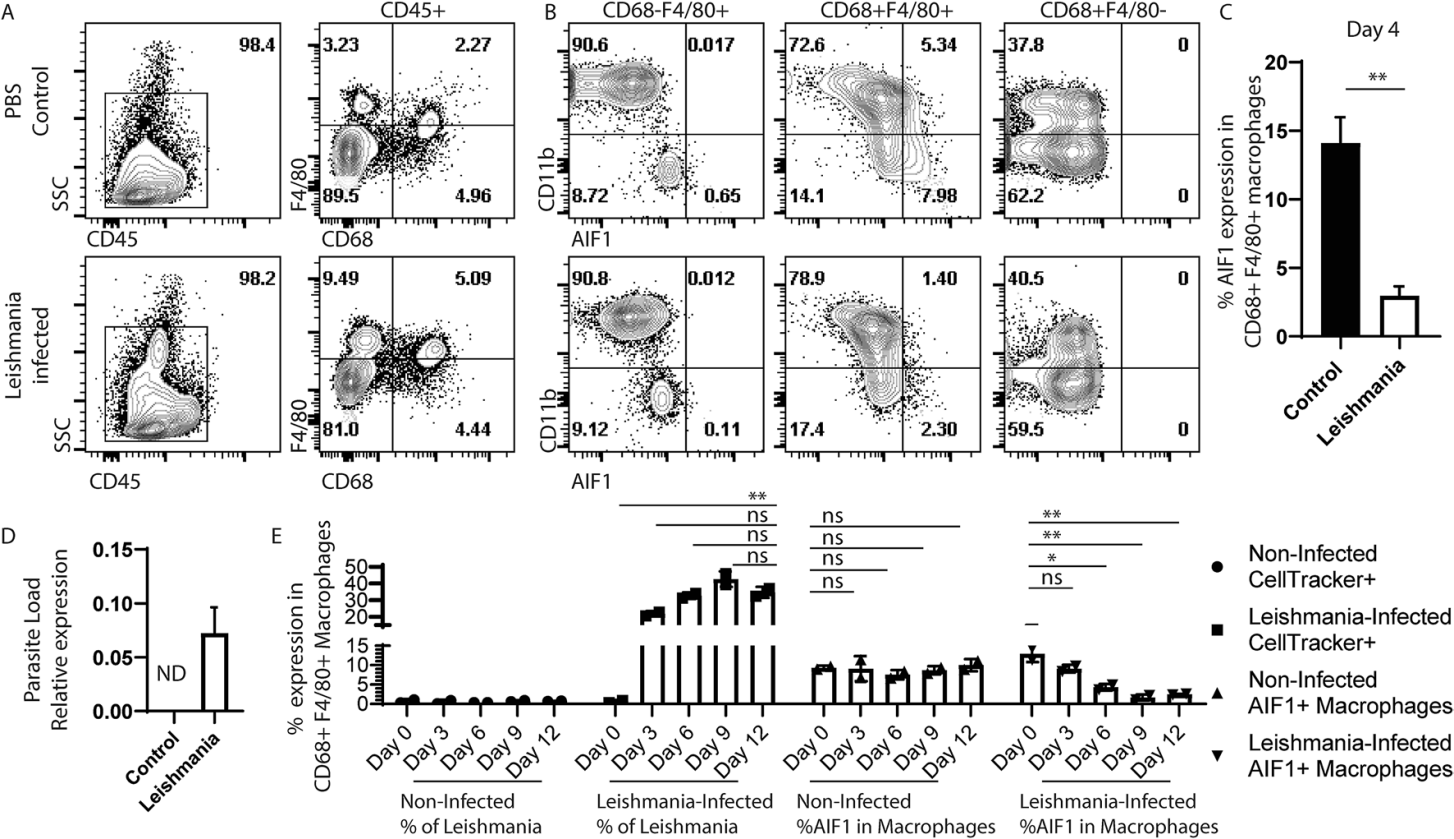
*Leishmania* infection correlates with lower number of AIF1^+^ macrophages in the spleen. C57BL/6 mice (*n* = 9) were intravenous injected with 10^6^ *Leishmania donovani* promastigotes. Control mice (*n* = 9) received PBS. 7 days after injection, spleen was collected from respective groups, each dissociated into single cell suspension and stained for flow cytometry. (**A**) Singlets were gated on CD45^+^ subsets to assess for F4/80 versus CD68 expression. (**B**) CD11b and AIF1 co-expression were assessed within each quadrant of the F4/80 versus CD68 populations in non-infected control versus *Leishmania donovani* infected groups. All gates were established using isotype controls. (**C**) Percentage of AIF1^+^ in the F4/80^+^CD68^+^ subsets is presented as a bar graph. (**D**) Parasite load within the spleen in control versus *Leishmania donovani* infected groups was measured by qPCR 7 days post-infection. Data shown as mean ± SEM representative of 3 independent experiments with 3 mice per group. (**E**) CellTracker-labeled *Leishmania donovani* parasites were injected into C57BL/6 mice (n = 10) and used to monitor frequency of infected macrophages every 3 days for 2 weeks. Control mice (n = 10) received PBS. Percentage of *Leishmania donovani* is determined by looking at frequency of CellTracker^+^ within CD11b^+/neg^F4/80^+^CD68^+^ macrophages of non-infected versus infected groups. Percentage of AIF1^+^ in CD11b^+/neg^F4/80^+^/CD68^+^macrophages was concomitantly determined in non-infected versus infected groups. Data shown as mean ± SEM representative of 2 independent experiments with 2 mice per group. Statistical significance was determined by unpaired *t*-test. *p < 0.05, **p < 0.01 and ns = not significant.

Prior studies have shown that *Leishmania* infections result in impaired macrophage functions and accumulation of Ly6C^+^ monocytes that failed to transition into terminally differentiated populations^7,8^. To evaluate whether AIF1 expression is altered during infections, wild type mice were in vivo challenged with *L. donovani* via intravenous administration. After 7 days of infection, spleens were harvested from infected and control groups prior to flow cytometric analyses. Results revealed increased frequency of F4/80^+^ subsets after infection, which is a marker largely used to identify monocytes and macrophages^25^ (Fig. 2A). AIF1 expression was predominately found within F4/80^+^CD68^+^CD11b^neg/lo^ splenic red pulp macrophages. However, in the *L. donovani*-infected cohorts, AIF1 expression within this subset was markedly decreased from 7.98% ± 2.1 to 2.30% ± 0.6 (Fig. 2B,C). Concomitantly, levels of *L. donovani* within the spleen were measured by qPCR to confirm successful infection (Fig. 2D). As follow-up experiments, relative amount of parasites and AIF1 expression within the splenic macrophages were assessed daily post-infection by flow cytometry. Percentages of CellTracker-labeled *L. donovani* present within the splenic pre-gated CD68^+^F4/80^+^ macrophages revealed increased loads relative to reduced AIF1 expression in the macrophages compared to the non-infected group controls (Fig. 2E). Notably, the increased replication of labeled parasites, particularly after the 9-day mark, can also contribute to the observed decreased percentage at later time points.

### Ectopic expression of AIF1 in macrophages restores anti-parasite immunity against *Leishmania*

**Figure 3:**
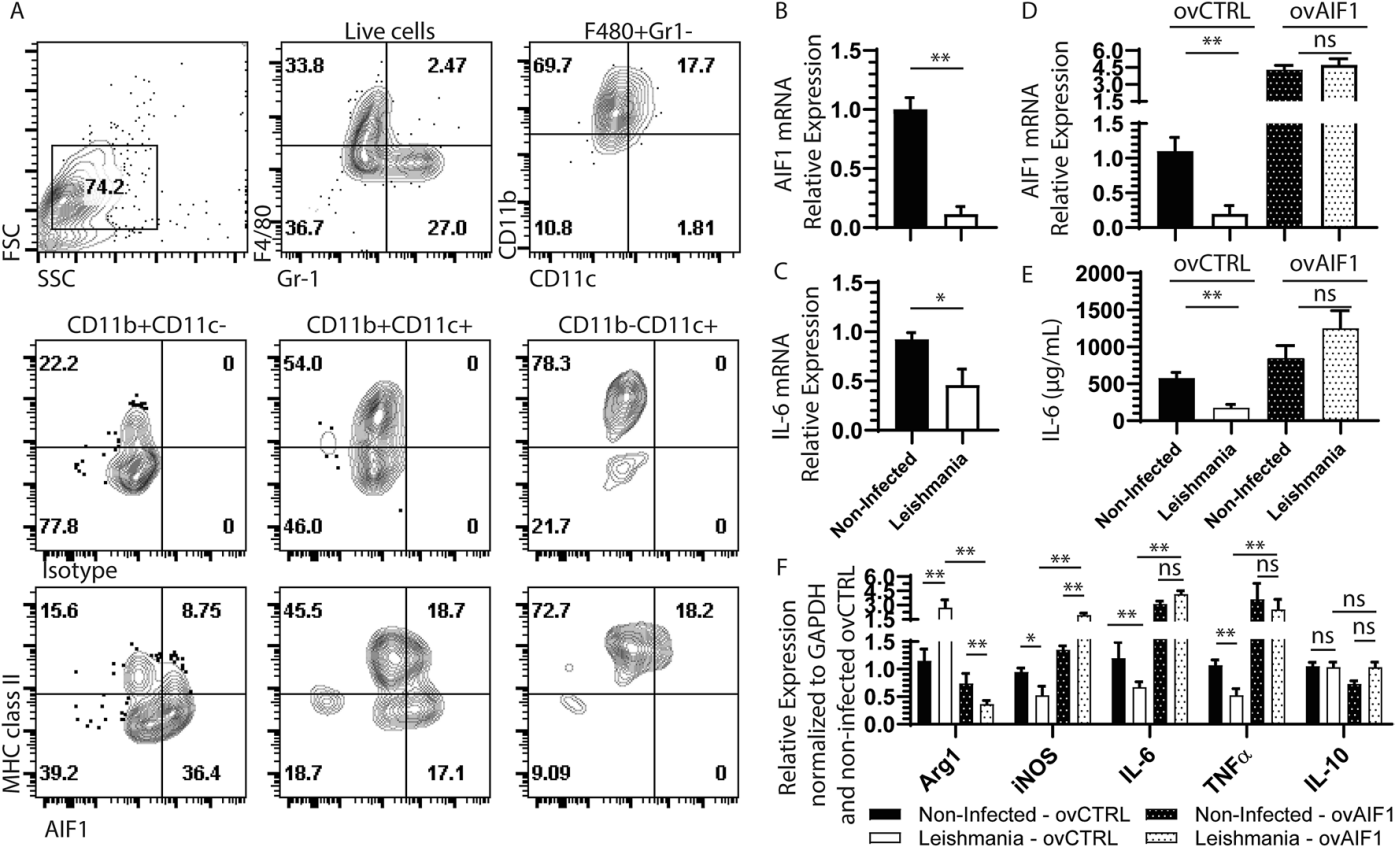
Bone marrow-derived macrophages (BMDM) express AIF1 under M-CSF stimulation. Bone marrow cells derived from wild type C57BL6 mice (*n* = 8) were differentiated into macrophages after 6 days of M-CSF stimulation. Differentiated cells were stained with Gr-1, F4/80, CD11b, MHC class II, CD11c and AIF1 prior to acquisition on a flow cytometric analyzer. (**A**) F4/80^+^Gr-1^neg^ cells were then assessed for expression of AIF1 in CD11b^+^CD11c^neg^, CD11b^+^CD11c^+^ and CD11b^neg^CD11c^+^. Isotype controls were used to establish the gating strategy for all quadrants. (**B**,**C**) For transcriptional analyses AIF1 and IL-6 was assessed upon in vitro infection with *L. donovani* in BMDM. Real-time PCR data is normalized to respective non-infected group controls. (**D**) Plasmid containing an overexpression vector for AIF1 (ovAIF1) under a non-repressible promoter was transfected into BMDM prior to infection with *L. donovani*. An empty expression vector served as control (ovCTRL). (**E**) IL-6 ELISA assays were performed on supernatant collected from ovAIF1 versus ovCTRL transfected BMDM infected with *L. donovani*. (**F**) Real-time PCR studies were performed to assess macrophage transcriptional expression changes for Arg1, iNOS, IL-6, TNFα, and IL-10 upon overexpression of AIF1 after *L. donovani* infection. Data are representative of four independent experiments with three replicates per group per experiment. Data shown as mean ± SEM. Statistical significance was determined by unpaired *t*-test. *p < 0.05 and **p < 0.01.

As in vivo experiments showed a correlation of *L. donovani* infection with reduced AIF1 expression, studies next assessed whether infection directly antagonized AIF1 using in vitromodels. To directly address, bone marrow-derived macrophages (BMDM) were employed to study the phenomenon. AIF1 expression was largely restricted to the CD11b^+^F4/80^+^Gr-1^neg^CD11c^neg^ BMDM subsets (Fig. 3A). In these committed macrophages, in vitro infection with *L. donovani* resulted in marked depression of AIF1, as assessed by real time PCR (Fig. 3B). This was in conjunction with reduced levels of IL-6 upon in vitro infection (Fig. 3C). Next, AIF1 was ectopically expressed under a non-repressible promoter in macrophages to assess impact on *L. donovani* infection (Fig. 3D). Results revealed that overexpression of AIF1 reduced parasite load (Supplemental Fig. 1) and supported increased levels of IL-6 cytokine production (Fig. 3E). Further transcriptomic analyses revealed that over expression of AIF1 inhibited Arg1 gene expression, had no effect on IL-10 and, in turn, resulted in increased iNOS and TNFα levels during *L. donovani* infection (Fig. 3F). Collectively, these results suggest that AIF1 is responsible for promoting pro-inflammatory responses and that *L. donovani* can directly antagonize these responses by inhibiting expression of the immunoregulatory gene.

### AIF1 is important for Ly6C^+^ monocytes conversion into macrophages

**Figure 4:**
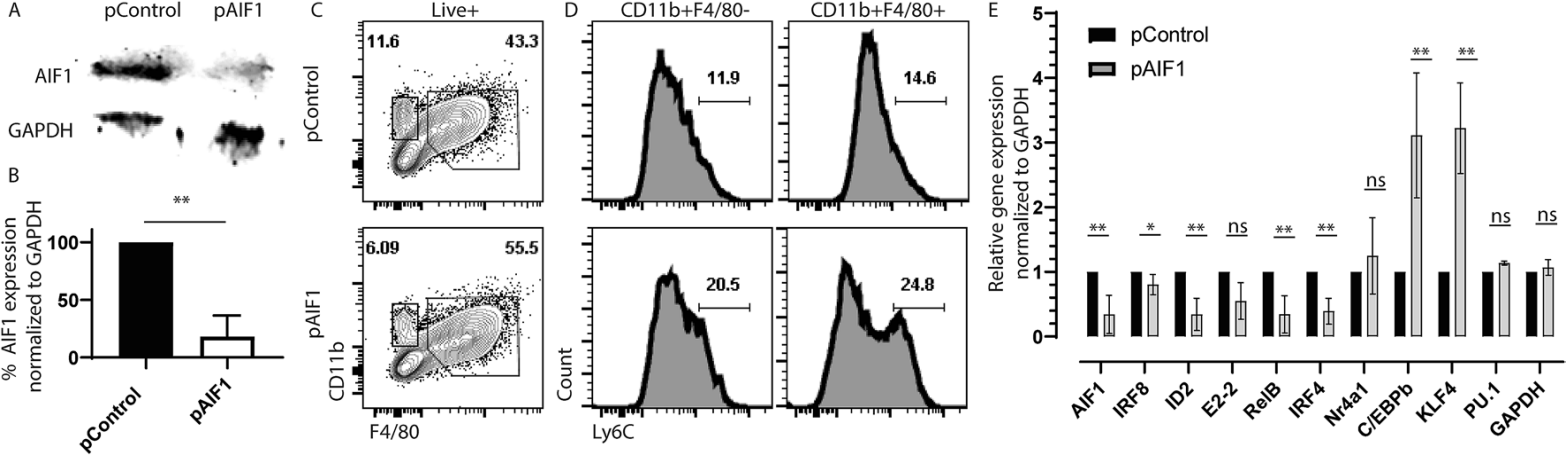
AIF1 silencing in bone marrow cells restrains macrophage differentiation. Bone marrow was collected from wild type mice prior to immediate transfection with Crispr-Cas9 plasmid carrying gRNA targeting AIF1 gene (pAIF1). Scramble gRNA served as control (pControl). Transfected bone marrow cells were stimulated for 6 days with M-CSF. (**A**) Western blot analysis was performed on pControl versus pAIF1 transfected bone marrow-derived macrophages to evaluate silencing of AIF1 expression. GAPDH served as internal loading control. Full-length blots/gels are presented in Supplementary Fig. 2. (**B**) Bar graph denotes percentage knockdown calculated using fluorescence intensity scale (ImageStudio 5.0). (**C**) For analogous studies, AIF1-silenced and control day 6 bone marrow-derived macrophages were collected and stained for CD11b, F4/80, and Ly6C prior to flow cytometric analysis. (**D**) Presence of Ly6C + monocytes were analyzed in both CD11b^+^F4/80^neg^ and CD11b^+^F4/80^+^ subsets. (**E**) Total RNA was isolated from AIF1-silenced or control bone marrow-derived macrophages differentiated under M-CSF stimulation for 6 days. Real-time PCR was performed to evaluate expression of: AIF1, IRF8, Id2, E2-2, RelB, IRF4, Nr4a1, C/EBPb, KL4 and PU.1. GAPDH was utilized as internal loading control. All data was normalized to the controls. Data shown as mean ± SEM and representative of at least three independent experiments. Statistical significance was determined by unpaired *t*-test. *p < 0.05 and **p < 0.01.

Prior studies have shown that *L. donovani* infection results in accumulation of Ly6C^+^ monocytes in the spleen^7^. Bone marrow infection by the parasite can suppress stromal macrophage functions and alter capacity to regulate hematopoiesis^26–28^. Notably, AIF1 is responsible for differentiation of monocytes into dendritic cells^12^. Therefore, to investigate the potential governing role of monocyte differentiation into macrophages, AIF1 was silenced in bone marrow cells prior to M-CSF stimulation in vitro. Transfection with Crispr-Cas9 plasmids carrying gRNA targeting AIF1 (pAIF1) consistently reduced protein expression down to 28% ± 9 compared to scrambled gRNA controls (pControl) (Fig. 4A,B). There was no change in viability or total number of cells upon silencing AIF1. However, flow cytometric analyses revealed an altered distribution of CD11b^+^F4/80^+^ and CD11b^+^F4/80^neg^ monocyte-macrophage populations within the AIF1 silenced versus control groups (Fig. 4C). Within both populations, silencing of AIF1 resulted in incomplete downregulation of Ly6C expression (Fig. 4D), whereby Ly6C^+^ subsets represent the monocyte pool and Ly6C^neg^ the macrophage groups. Lastly, transcriptomic profiling revealed increased expression of essential monocyte genes C/EBPb and KLF4, with a concomitant decrease in ID2, RelB and IRF4 (Fig. 4E). The expression of the genes PU.1 and Nr4a1 were not affected. Further studies revealed no alteration in p38, ERK1/2, IkB, p65 and p52/p100 phosphorylation signaling cascades (data not shown).

**Figure 5:**
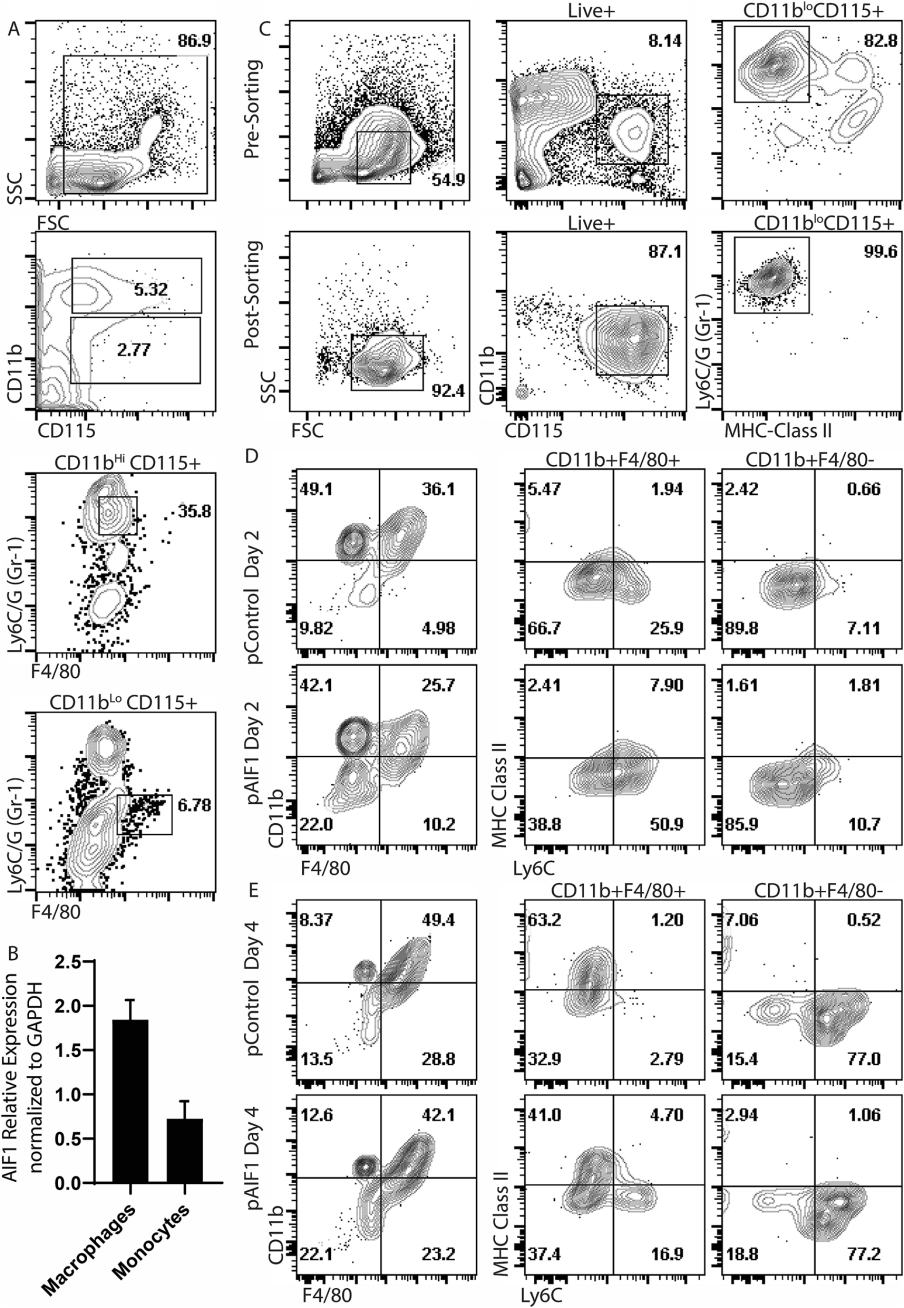
Ly6C^+^ sorted monocytes require AIF1 to differentiate into macrophages. (**A**) CD11b^hi^CD115^+^Ly6C/G^+^F4/80^neg^ monocytes and CD11b^lo^CD115^+^Ly6C/G^neg^F4/80^+^macrophages were flow cytometric sorted from the spleen of wild type C57BL/6 mice (*n* = 3). (**B**) AIF1 gene expression was then assessed by real-time PCR in the monocytes versus macrophage populations. (**C**) Bone marrow cells isolated from C57BL/6 (*n* = 6) are shown prior to (pre-sorting) and after sorting (post-sorting) to purify CD11b^low^CD115^+^Ly6C/G^+^F4/80^neg^ monocytes. (**D**) Isolated CD11b^low^CD115^+^Ly6C/G^+^F4/80^neg^ monocytes were then immediately silenced for AIF1 prior to culturing with M-CSF. After (**D**) 2 days and (**E**) 4 days of M-CSF stimulation, cells were assessed for Ly6C and MHC class II expression within the CD11b^+^F4/80^+^ and CD11b^+^F4/80^neg^ populations. Data shown as mean ± SEM and representative of three independent experiments. Statistical significance was determined by unpaired *t*-test. *p < 0.05 and **p < 0.01.

To further evaluate role of AIF1 in monocyte-macrophage lineages, monocytes (CD11b^hi^CD115^+^Ly6C/G^+^F4/80^low^) and macrophages (CD11b^low^CD115^+^Ly6C/G^neg^F4/80^+^) were FACS-sorted from spleen^29,30^ (Fig. 5A). Gene expression analyses revealed higher levels of AIF1 in splenic macrophages compared to the monocytes (Fig. 5B). Given moderate levels of AIF1 expression in monocytes, studies next evaluated whether AIF1 expression in monocytes is important for directing differentiation into macrophages ex vivo. CD11b^hi^CD115^+^Ly6C/G^+^MHC class II^neg^ monocytes were FACS-sorted from bone marrow prior to Crispr-Cas9-mediated silencing of AIF1 (Fig. 5C). Monocytes were then cultured for 4 days under M-CSF stimulation. On day 2 under M-CSF stimulation, there was a notable twofold increase in Ly6C expression from AIF1-silenced groups compared to controls in the CD11b^+^F4/80^+^ population (Fig. 5D). This was also concomitant with higher levels of MHC class II in the CD11b^+^F4/80^+^ subsets upon AIF1 silencing; F480^+^Ly6C^+^MHC class II^+^ population has been shown to represent tissue monocytes^31^. By day 4 within the CD11b^+^F4/80^+^ population, there was 16.9% ± 2.7 Ly6C^+^ MHC class II^neg^ subsets in AIF1-silenced cohort compared to only 2.79% ± 0.6 in control groups stimulated with M-CSF (Fig. 5E). Furthermore, there was a significant reduction in Ly6C^neg^ MHC class II^+^ subsets in absence of AIF1. Taken together, these studies suggest that in absence of AIF1, subsets of ex vivo-isolated monocyte are restrained from conversion into macrophages under M-CSF stimuli.

## Discussion

The major aim of these studies was to evaluate the role of AIF1 in combating *Leishmania* parasitic infections. Prior reports have shown that *L. donovani* effectively evades immunity, marked by depressed macrophage pro-inflammatory response and accumulation of poorly functioning inflammatory monocytes within the spleen. In this report, findings reveal that *L. donovani* infections in mice correlated with a reduction in AIF1 expression in myeloid cells. However, these findings cannot concretely distinguish whether *Leishmania* infection triggered infiltration of myeloid cells not yet expressing AIF1 or whether the infection does directly result in downregulation of AIF1 expression. This is particularly pertinent given that monocytes express relatively lower levels of AIF1 expression, and levels are upregulated upon conversion into splenic macrophages. Follow-up in vitro studies did reveal that the parasite can directly antagonize AIF1 expression, which was associated with depressed clearance of pathogen load and impaired monocyte-to-macrophage differentiation. Furthermore, overexpression of AIF1 in *Leishmania-*infected macrophages in vitro retained elevated IL-6 and TNF-α responses, countering the effect of the parasite on inhibition of immune effector responses. The result also led to reduced Arg1 expression concomitant with increased iNOS levels. Although several studies have highlighted the importance of AIF1 in a variety of other diseases, this is the first report to show the importance of the gene in monocyte-macrophage immune responses and presents a model whereby *L. donovani* can evade host immunity by inhibiting AIF1.

Splenic red pulp macrophages, defined as CD11b^lo/neg^F4/80^hi^CD68^+^, as well as bone marrow derived CD11b^+^F4/80^+^CD68^+^CD11c^neg^ macrophages generated in vitro under M-CSF stimuli, express high levels of AIF1. Care was employed during flow cytometric analyses to compensate for intrinsic differences in autofluorescence among subsets^32^. Infection of mice with *L. donovani* in vivo or in vitro infection using BMDM approaches had a correlative reduction in frequency of AIF1^+^ macrophages. To concretely assess the role of AIF1 in directing immune responses, Crispr-Cas9-mediated silencing of AIF1 in macrophages and monocytes corroboratively revealed suppressed immune responses. Notably, silencing of AIF1 expression within bone marrow-derived cells allowed differentiation up through the Ly6C^+^ monocyte stage in vitro under M-CSF stimuli. However, movement beyond Ly6C^+^ monocytes into terminally differentiated macrophages was partially inhibited. This follows suit with literature reports of increased frequency of Ly6C^+^ monocytes subsets upon *L. donovani* infection^7^. Furthermore, silencing of AIF1 in sorted Ly6C^+^ monocytes resulted in impaired conversion to macrophages, marked by increased frequency of CD11b^+^F4/80^−^ subsets. This may suggest that AIF1 is further involved in reinforcing the inflammatory state of monocytes and that loss regresses the committed differentiation state. Future studies will delineate in vivo how loss of AIF1 in the context of steady-state versus inflammatory settings can affect total cell numbers of monocytes versus macrophages, or whether there is solely a disruption in altered frequency distribution (i.e. monocyte-to-macrophage conversion).

Thus, for establishing a foothold for disease, inhibiting macrophage responses, restricting monocyte-to-macrophage differentiation and disrupting circulating inflammatory monocyte antiparasitic functions are necessary for parasite survival. Thus, *L. donovani* suppression of AIF1 would serve as an effective immune evasion strategy and the premise does support extensive published work^3,7,11,34^. This is particularly relevant as prior studies have shown that suppression of AIF1 in dendritic cells redirects cognate responder naïve T cells towards a regulatory phenotype^13,14^. Additional studies have shown that suppression within hematopoietic progenitors or monocytes impairs ability to differentiate into conventional DC and monocyte-deriv^33^ed DC (under GM-CSF stimuli), respectively^12^.

Mechanistically, *L. donovani* infection has been shown to disrupts PKC downstream signaling activities by interfering with binding of Ca^2+^, which were determined to be defects in kinase activation (as opposed to lowering of total levels of PKC)^35–37^. This is a particular important and relevant finding, given that AIF1 has been shown to interact directly with PKC to promote both differentiation and effector immune responses in myeloid cells^12^. Taken together, this works supports the notion that *Leishmania* parasites can effectively evade immune responses by inhibiting expression of AIF1 to antagonize downstream signaling cascades in monocyte-macrophage lineages, which thereby restrains anti-parasitic immunity. Future studies warrant delineating the intracellular signaling mechanisms that allow *Leishmania* parasites to directly repress AIF1 expression. Knowledge gained will help to develop small molecule inhibitors that can disrupt *Leishmania* suppression of AIF1 and thereby increase health outcomes of infected individuals.

## Materials and methods

### Animals

Mice were purchased from The Jackson Laboratory (Bar Harbor, ME) and housed in pathogen-free facilities at Howard University. C57BL/6 (wild type; WT) male and female mice 8–12 weeks of age were used as a source of bone marrow and spleen. WT mice were additionally used for in vivo challenge experiments. All animal procedures were performed in accordance and approved by the Institutional Animal Care and Use Committee through the Office of Regulatory Research and Compliance at Howard University.

### Generation of bone marrow-derived macrophages and monocytes

Macrophages and monocytes were generated from isolated murine bone marrow cells. Briefly, bone marrow cells from mouse tibias and femurs were passed through a 70 μm nylon mesh to remove debris prior to culturing with RPMI (Thermo Fisher; Grand Island NY) supplemented with 10% fetal bovine serum (FBS; Gibco), 100 U/mL penicillin/streptomycin (Gibco) and 20 ng/mL M-CSF (Peprotech; Rochy Hill NJ), or the equivalent of L929 cell line supernatant, for 6 days in culture. Cells were confirmed macrophages by flow cytometric analyses for CD11b^+^F4/80^+^CD115^+^Ly6C/G^neg^CD11c^neg^ and monocytes for CD11b^+^CD115^+^Ly6C^+^ markers.

### Sorting of Ly6C^+^ monocytes and terminally differentiated F4/80^+^ macrophages

Spleens harvested from wild type mice were dissociated using the GentleMACS dissociator (Miltenyi) prior to red blood cell lysis treatment and staining with antibodies to MHC class II, Ly6C/G, CD115 (M-CSF receptor), and CD11b. All antibodies were purchased from BioLegend. Cells were then sorted for CD11b^hi^ Ly6C/G^+^ CD115^+^ MHC class II^neg^ using the BD FACSJazz flow cytometric sorter (BD Biosciences). For macrophage isolation, spleens were dissociated using GentleMACS prior to red blood cell lysis treatment and staining with antibodies to CD11b, Ly6C/G, F4/80 and MHC class II. Cells were then sorted for CD11b^lo^, F4/80^+^, Ly6C/G^neg^, MHC class II^+^.

### *Leishmania* parasites infection

*Leishmania donovani* promastigotes (strain MHOM/IN/80/DD8) were obtained from ATCC. For growth and expansion, parasites were inoculated into Novy, Mac Neal and Nicole (NNN) and Schneider's Insect medium (Thermo Fisher) supplemented with 10% heat-inactivated fetal bovine serum and 1% penicillin/streptomycin. Parasites were kept in frozen stocks after only one passage in culture to limit expansion of mutant strains. *Leishmania* isolates were expanded in supplemented Schneider’s medium at 24 °C. Promastigotes were examined daily using light microscopy to determine growth curves. For in vivoinfections, 10-week old mice were infected by intravenous injection of 10^6^ stationary-phase parasites in 200 μl of warm PBS^38^. In some studies, *Leishmania* parasites were pre-labeled with CellTracker staining dye (Thermo Fisher). Intracellular parasite load within cells was determined by pre-gating on target macrophage populations prior to evaluating percentage of CellTracker^+^ *Leishmania* parasites present by flow cytometry. The approach follows published methods by Silva et al.^1^. Dilution of the dye due to parasite in vivo replication allows for up to 2 weeks for tracking. Control mice received PBS only. Infection was allowed to proceed for 7–14 days. Blood and tissues of the spleen, liver and lungs were harvested after mice were sacrificed; sera were prepared from whole blood. For in vitro infections, bone marrow cells, monocytes or macrophages were infected at a 5:1 ratio of promastigotes to cells prior to incubation for 48–72 h. Parasite load was quantified by preparing DNA from tissues or culture samples prior to performing real-time PCR using a modified approach by Wilson et al^39^.

### Antibodies and live/dead staining dye

AIF1 (EPR16588) antibody was purchased from Abcam (Cambridge MA). CD117 (2B8), CD11c (N418), MHC class II (M5/114.15.2), CD11b (M1/70), F4/80 (BM8), CD135 (A2F10), CD45 (30-F11), CD68 (FA-11), CD64 (X54-5/7.1), Ly6C (HK1.4), and Gr-1 (RB6-8C5) antibodies were all purchased from BioLegend (San Diego CA). GAPDH (GA1R) antibody and Live/Dead fixable dead cell stain were purchased from Thermo Fisher.

### CRISPR-mediated gene silencing, small interfering RNA and electroporation

The CRISPR Cas9 system was used to knockout AIF1 in total BM or CD11b^+^Ly6C/G^+^CD115^+^MHC class II^neg^. The CRISPR DNA plasmid was created using the GeneArt CRISPR Nuclease Vector Kit (Thermo Fisher) to target AIF1, as previously described by Elizondo et al^12^. The gRNA sequences are: 5′-GCTGAAGAGATTAATTAGAG-3′ (pAIF1). Control plasmids contained scrambled targeting sequences (pControl). Plasmids were purified using PureLink HiPure Plasmid Maxiprep Kit (Thermo Fisher) and cleaned with the MiraCLEAN Endotoxin Removal Kit (Mirus, Madison WI). Cells were transfected with 40 μg of control or AIF1 targeting plasmids using a square wave electroporator with the following parameters: 230 V, 4 ms, 5 pulses. For small interfering RNA (siRNA)-mediated silencing of cells, 0.5 nmol of the oligonucleotide sequence 5′-GGCAAGAGAUCUGCCAUCUUG-3′ was electroporated under the following settings: 310 V, 10 ms, 1 pulse.

### Cryosectioning and fluorescence microscopy

Spleens were fixed and cryosectioned prior to transferring onto L-poly Lysine-coated glass slides. 0.3% Triton-X solution was used to permeabilize the sections followed by blocking with 0.2% BSA. Sections were stained with CD11b, CD64, F4/80, AIF1 or IgG isotype control antibodies. DAPI (Thermo Fisher) was used as a nuclear staining dye. Slides were imaged using the FSX100 fluorescence microscope (Olympus, Waltham MA). Acquired images were then analyzed using ImageJ (Rasband, W.S., ImageJ, U.S. National Institutes of Health) and FlowJo (Flow Jo LLC; Ashland, OR).

### Gene expression profiling by quantitative PCR (qPCR)

To evaluate gene expression, cells were harvested and resuspended in Trizol (Thermo Fisher) prior to total RNA extraction. Total RNA was reverse transcribed into single-stranded cDNA using the High Capacity cDNA Reverse Transcription Kit (Thermo Fisher). For quantitative PCR reactions, Gene Expression TaqMan Fast Advanced Master Mix (Cat# 4444557), and the following probes purchased from Thermo Fisher were used: AIF1 (Mm00479862_g1), RelB (Mm00485664_m1), ID2 (Mm00711781_m1), IRF8 (Mm00492567_m1), IRF4 (Mm00516431_m1), Nr4a1 (Mm01300401_m1), C/EBPb (Mm00843434_s1), KLF4 (Mm00516104_m1), PU.1 (SPIB1;Mm00488140_m1), IL-6 (Mm00446190_m1) and IL-10 (Mm01288386_m1). For *L. donovani*, the following probes were used: Forward-GCGGTGGCTGGTTTTAGATG, Reverse-TCCAATGAAGCCAAGCCAGT and Taqman primer sequence CCCATACCACCAAACGCAGCCCA^39^. Samples were analyzed on the QuantStudio 5 real time-PCR system (Thermo Fisher). Expression levels of the target transcripts were calculated by the comparative Ct method (2^–ΔΔCt^ formula) after normalization with the housekeeping gene GAPDH (Mm99999915_g1) or ACTB (Mm02619580_g1).

### Flow cytometry

For in vitro cell culture experiments, cells are isolated from plates using physical scraping approaches. Tissue isolation procedures employed the GentleMACS dissociator (Miltenyi) in PBS; no trypsin or collagenase treatments were used. Single suspension of cells were washed with PBS supplemented with 1 mM EDTA and stained with fluorochrome-labeled antigen-specific antibodies or respective isotype controls. For intracellular antibody labeling, cells were fixed in 3% PFA prior to permeabilizing with 0.2% saponin in PBS and staining with antibodies. Cells were then acquired on a BD FACSVerse flow cytometric analyzer (BD Biosciences) and data analyzed using FlowJo (FlowJo LLC; Ashland, OR). All gates for dot plots and histograms were established using appropriate isotype controls.

### Western blot analysis

Lysates were prepared using NP-40 lysis buffer supplemented with a protease-phosphatase inhibitor cocktail (IBI Scientific). Lysates were ran on a 10% SDS-PAGE gel prior to transferring to nitrocellulose blots using semi-dry transfer system (PowerBlotter; Thermo Fisher). Membranes were stained with primary antibody prior to wash and secondary antibody labeling. Protein bands within blots were then detected using the Licor Odyssey imaging system (Licor, Lincoln NB) and analyzed using Image Studio 5.2 software (Licor).

### ELISA

Supernatant from in vitro and ex vivo infected cells were collected and stored at − 80C prior to analyses. Similarly, sera from infected mice were collected and stored at − 80C until use. Levels of IL-6 and TNFα were measured using ELISA kits (BioLegend) following manufacture recommended protocol.

### Statistical analysis

GraphPad Prism v8.0 (GraphPad Software, La Jolla CA) was used to determine statistical significance. Student unpaired two-tailed t-test was used to evaluate the significance between two groups. Error bars for all figures indicate standard errors; *< 0.05, **< 0.01 and NS = not significant.

## Supplementary information

Supplementary Figures.

## Abbreviations

AIF1: Allograft Inflammatory Factor-1
BMDM: Bone marrow-derived macrophages
DC: Dendritic cells
MoDC: Monocyte-derived dendritic cells
PKC: Protein kinase C
VL: Visceral leishmaniasis
