# Supplementary material for: Leishmania donovani infection suppresses Allograft Inflammatory Factor-1 in monocytes and macrophages to inhibit inflammatory responses
Source: Sci Rep. 2021 Jan 13;11:946. doi: 10.1038/s41598-020-79068-6 (PMC7807085; doi:10.1038/s41598-020-79068-6)
Supplement: Supplementary file 1 — Supplementary Figures. [file 41598_2020_79068_MOESM1_ESM.pdf]

Leishmania donovani infection suppresses Allograft Inflammatory Factor-1 in monocytes and macrophages to inhibit inflammatory responses

Ricardo L. da Silva[1,2], Diana M. Elizondo[1,3], Nailah Z. D. Brandy[1], Naomi L. Haddock[1,4], Thomas A. Boddie[1], Lais Lima de Oliveira[2], Amélia R. Jesus[2], Roque P. Almeida[2], Tatiana R. de Moura[2], and Michael W. Lipscomb[1]

[1]Department of Biology, Howard University, Washington, DC, USA
[2]Laboratory of Immunology and Molecular Biology, Federal University of Sergipe, Aracaju, Brazil
[3]Department of Pediatrics and Communicable Diseases, Univ of Michigan Medical School, Ann Arbor, MI, USA
[4]Stanford Immunology, Stanford University, Stanford, CA USA.

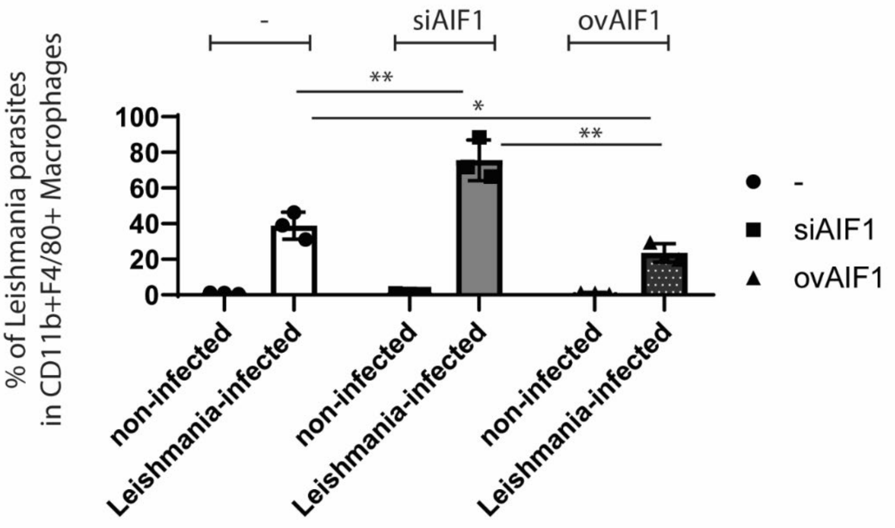

Supplemental Figure 1. Amount of *Leishmania Donovani* in bone marrow-derived macrophages. Macrophages were either untreated (-), silenced for AIF1 (siAIF1), or overexpressed for AIF1 (ovAIF1) prior to being infected with CellTracker-stained *Leishmania donovani* at 5 parasites to 1 macrophage ratio. Next, percentage of $CellTracker^{+}$ Leishmania co-localized within $CD11b^{+}F4/80^{+}$ macrophages was measured by flow cytometry after 48 h. Data represents 2 independent experiments by 3 replicates per group and presented as mean ±SEM. Statistical significance was determined by unpaired t-test. * = p<0.05 and ** = p<0.01.

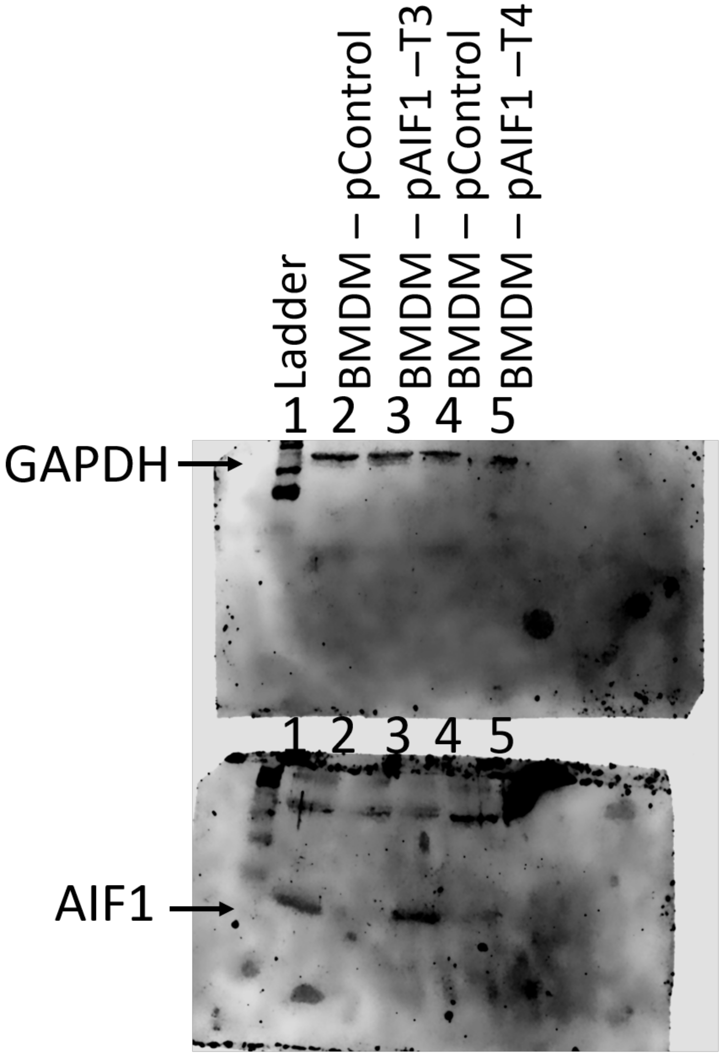

Supplemental Figure 2. Full length uncropped gel from Figure 4. Lane 1: Ladder. Lane 2-5: Bone marrow-derived macrophages for 6 days with M-CSF stimuli and transfected with either (2) pControl-T3-Scramble, (3) pAIF1-T3, (4) pControl-T4-Scramble or (5) pAIF1-T4. T3 and T4 have different gRNA targeting AIF1 gene exons.
